# Multislice Computed Tomography Assessment of Airway Patency Changes Associated with Mandibular Advancement Appliance Therapy in Supine Patients with Obstructive Sleep Apnea

**DOI:** 10.1155/2019/8509820

**Published:** 2019-03-03

**Authors:** Yu Matsumura, Hiroshi Ueda, Toshikazu Nagasaki, Cynthia Concepción Medina, Koji Iwai, Kotaro Tanimoto

**Affiliations:** ^1^Department of Orthodontics and Craniofacial Developmental Biology, Graduate School of Biomedical Science, Hiroshima University, 1-2-3 Kasumi, Minami-ku, Hiroshima 734-8553, Japan; ^2^Department of Oral and Maxillofacial Radiology, Graduate School of Biomedical Science, Hiroshima University, 1-2-3 Kasumi, Minami-ku, Hiroshima 734-8553, Japan

## Abstract

The purpose of the present study was to measure the regional effects of the mandibular advancement appliance (MAA) on the upper airway of supine subjects with obstructive sleep apnea (OSA) using multislice computed tomography (MSCT). The subjects included 8 males and 5 females who were diagnosed with mild to moderate OSA and were referred to the Orthodontic Clinic of Hiroshima University Hospital, where they underwent MAA therapy. Using a CT scanner, baseline MSCT images were obtained from the subjects without the MAA for morphological analysis, and then the experimental images were obtained while wearing the MAA. To measure the anteroposterior diameter, width, and cross-sectional area of the oropharynx region of interest (ROI), five distance variables were first defined on each multiplanar reconstruction (MPR) image using OsiriX. Additionally, the volumes of the upper airway, bony hard tissue, and soft tissue (soft palate and tongue) in the oro-hypopharyngeal region were measured. In most of the assessed airway size variables, significant increases in the anteroposterior diameter and width were observed after MAA therapy. Regarding the upper airway cross-sectional area, all the upper airway size variables exhibited significant increases. In the volumetric analysis, a significant increase was observed in airway volume, whereas the soft tissue volume in the oro-hypopharyngeal region did not show the significant decrease after MMA therapy. However, from a different point of view, the volumes of the upper airway and soft tissue significantly increased and decreased, respectively, as demonstrated by the calculated ratio for the oro-hypopharyngeal region. We demonstrated that the proportional size of the soft tissue volume, i.e., the soft palate and tongue in the oro-hypopharyngeal region, significantly decreased during use of an MAA. This forward displacement of the soft tissue thereby increases the retroglossal airway space (except the nasopharynx) three-dimensionally.

## 1. Introduction

Obstructive sleep apnea (OSA) is characterized by periodic reduction or cessation of breathing due to narrowing of the upper pharyngeal airway during sleep. It is a common condition with significant public health consequences because of its association with morbidity [1] and mortality [2]. Use of the mandibular advancement appliance (MAA) has been established as a lifelong treatment for patients with mild to moderate OSA and those with severe OSA who refuse nasal continuous positive airway pressure (CPAP) therapy [3, 4]. The aim of MAA therapy is to enlarge the pharyngeal airway by repositioning the mandible forward, and MAAs have been shown, via cephalometric radiographs, to increase various upper airway dimensions in patients when they are awake [5–7].

Recent studies of upper airway size assessment have used three-dimensional data, such as that obtained with cone-beam computed tomography (CBCT). CBCT scanning accurately determines the cross-sectional area of the upper airway; images are obtained only in the axial plane, but can be reconstructed for volumetric analysis. Previous CBCT studies have documented the assessment of pharyngeal airway morphology with subjects in the upright position [8–10]. However, OSA is characterized by recurrent upper pharyngeal airway obstruction during sleep in the supine position.

Therefore, the purpose of the present study was to measure the regional effects of the MAA on the upper airway of supine subjects with OSA using multislice computed tomography (MSCT).

## 2. Materials and Methods

### 2.1. Subjects

The subjects included 8 males and 5 females (mean age, 48.8 +/- 17.6 years; body mass index, 22.4 +/- 3.8) who were diagnosed with mild to moderate OSA and were referred to the Orthodontic Clinic of Hiroshima University Hospital, where they underwent MAA therapy. The inclusion criteria were as follows: (1) mild to moderate OSA, (2) no severe facial skeletal discrepancies, (3) no periodontal disease, (4) no temporomandibular disorder (TMD) symptoms, and (5) no complications from serious systemic disease. The Ethics Committee of Hiroshima University Hospital approved this study's protocol, and informed consent was obtained from each subject prior to treatment (E-57).

### 2.2. Mandibular Advancement Appliance

**Figure 1:**
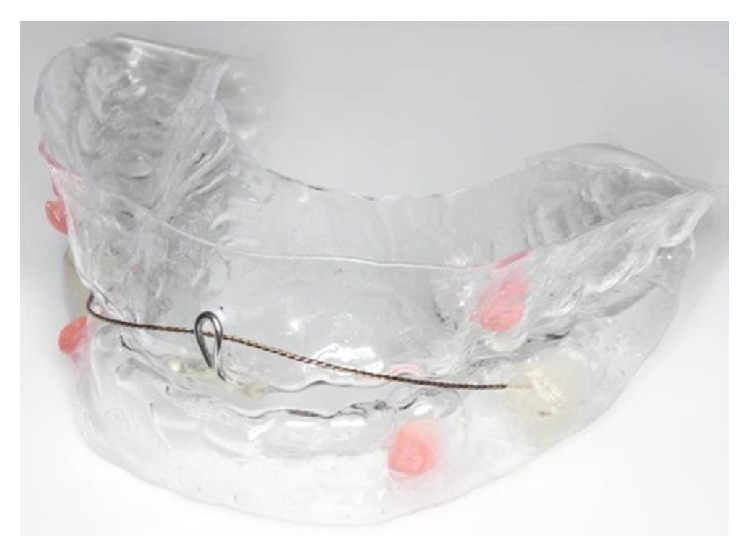
The mandibular advancement appliance.

The appliance consisted of two occlusal splints (i.e., one upper/maxillary and one lower/mandibular) held together by an orthodontic wire (Figure 1). The splints were constructed using 0.75-mm thick acrylic resin that provided full occlusal coverage of the teeth. A 0.0215-inch multistranded, twisted wire was attached on the buccal side of the lower splint. Patients could easily connect the lower splint to the upper splint. Initial mandibular advancement was defined as the edge to edge bite position (mean advancement: 3.4 +/- 2.7 mm) with a 3–4 mm vertical opening between the upper and lower anterior teeth [11].

### 2.3. Radiographic Examination

Using an Aquilion ONE^TM^ CT scanner (Toshiba Medical Systems, Tochigi, Japan), baseline MSCT data were obtained from the subjects without the MAA for morphological analysis, and then the experimental data were obtained while wearing the MAA. The MSCT scanning parameters were as follows: 80 detector-row helical scan, 240 x 240-mm field of view, 0.5-mm slice thickness, 512 x 512 matrix, 120 kV, and 100 mA. All patients were positioned in the Frankfurt horizontal (FH) plane, perpendicular to the floor with the facial midline parallel to the floor, and were asked to maintain this resting body position. The subjects were instructed to take a deep breath twice and to swallow once. After 5 s, the MSCT scans were performed. OsiriX ver 3.9 (Pixmeo SARL, Geneva, Switzerland) was used to analyze the digital imaging and communications in medicine (DICOM) files that were extracted from the MSCT data. The individual DICOM files were opened in OsiriX, and multiplanar reconstruction (MPR) was used to obtain cross-sectional slices, which were verified for landmark location and anatomic contours by the first author (Y. M.).

### 2.4. Measurements

**Figure 2:**
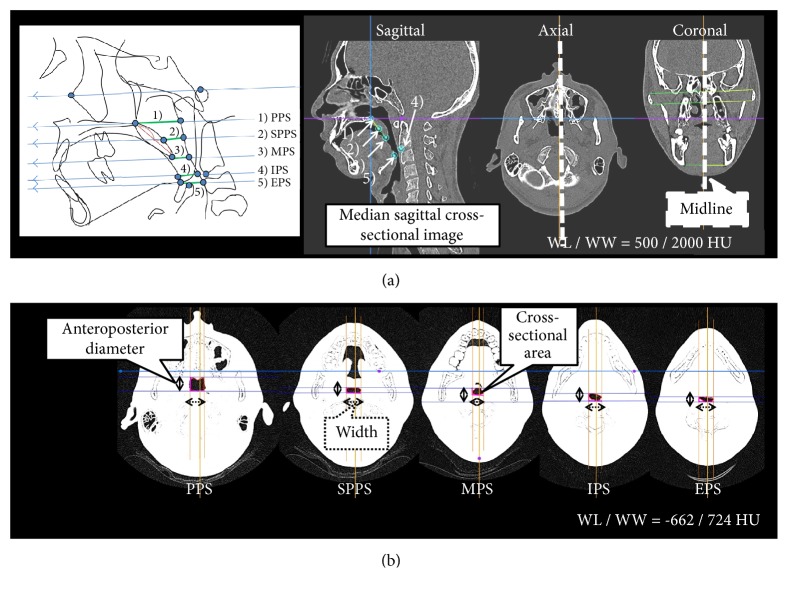
The measurement variables. (a) Midline and median sagittal cross-sectional images. (b) The five measurement sites of the upper airway on a multiplanar reconstruction (MPR) image. The multislice computed tomography data were imported into OsiriX ver. 3.9, with the window level/window width (WL/WW) set to the CT-bone value of 500/2000 HU. Slice datasets were coordinated as follows: (1) the midline of a midpoint of two straight lines, connecting the uppermost and lowermost ends of the bilateral zygomatic process observed in the coronal section, and a straight vertical line parallel to that midpoint were drawn; (2) the median sagittal cross-sectional image was a sagittal section sliced by the midline, and the median sagittal and axial cross-sections were set to the posterior edge of the maxillary bone (PNS); (3) five distance points were established on the MPR image by verifying on the median sagittal, axial, and coronal cross-sectional images; and (4) the WL/WW was set to the airway component value of -662/724 HU, and the anteroposterior diameter, width, and cross-sectional area at the five distance points were measured on the axial cross-sectional images. Abbreviations: EPS, epiglottic pharyngeal space; IPS, inferior pharyngeal space; MPS, middle pharyngeal space; PPS, palatal pharyngeal space; SPPS, superior posterior pharyngeal space.

To measure the anteroposterior diameter, width, and cross-sectional area of the oropharynx region of interest (ROI), five distance variables (cm/cm^2^) were first defined on each MPR image using OsiriX (Figure 2(a)). These measurement variables were chosen in accordance with those defined by Uozumi et al. [12]:

1. Palatal pharyngeal space (PPS): the anteroposterior depth of the pharynx measured between the posterior pharyngeal wall and the posterior nasal spine (PNS) on a line parallel to the FH plane that runs through the PNS.
2. Superior posterior pharyngeal space (SPPS): the anteroposterior depth of the pharynx measured between the posterior pharyngeal wall and the dorsum of the soft palate on a line parallel to the FH plane that runs through the middle of the line from the PNS to the tip of the soft palate.
3. Middle pharyngeal space (MPS): the anteroposterior depth of the pharynx measured between the posterior pharyngeal wall and the dorsum of the tongue on a line parallel to the FH plane that runs through the tip of the soft palate.
4. Inferior pharyngeal space (IPS): the anteroposterior depth of the pharynx measured between the posterior pharyngeal wall and the surface of the tongue on a line parallel to the FH plane that runs through the most anterior point of the second cervical vertebra.
5. Epiglottic pharyngeal space (EPS): the anteroposterior depth of the pharynx measured between the posterior pharyngeal wall and the surface of the tongue on a line parallel to the FH plane that runs through the tip of the epiglottis.

**Figure 3:**
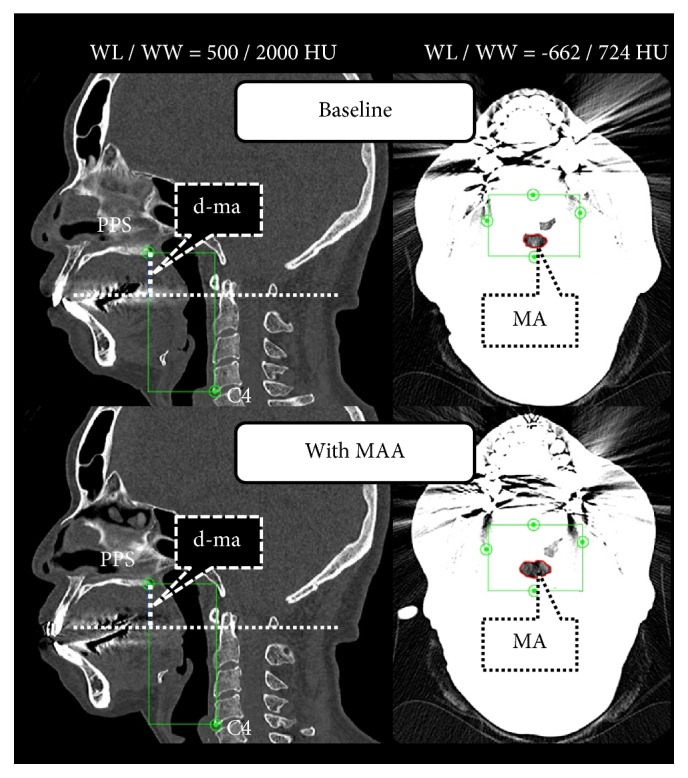
The minimum area (MA) on multiplanar reconstruction (MPR) images. The slice datasets were coordinated as follows: (1) The median sagittal cross-sectional image was set by using the baseline (T1) data (window level/window width [WL/WW] was set to the CT-bone value of 500/2000 HU). (2) The craniocaudal direction was set with the upper end as the palatal pharyngeal space (PPS) and the lower end as the lowermost anterior point of the fourth cervical vertebrae (C4) on the MPR image by verifying on the median sagittal, axial, and coronal cross-sectional images. (3) The WL/WW was set to the airway component value of -662/724 HU, the site where the cross-sectional area within that range was the narrowest by verifying on the axial cross-sectional image was set as the MA, and the anteroposterior diameter, width, and cross-sectional area were measured. (4) The WL/WW was again set to the CT-bone value of 500/2000 HU, and the vertical distance (d-ma) from the PPS to the MA was measured on the median sagittal cross-sectional image. (5) The median sagittal cross-sectional image was set using data with the mandibular advancement appliance (MAA), and each d-ma obtained from the baseline data was reproduced as multislice computed tomography (MSCT) data with MAA. (6) The WL/WW was set to the airway component value of -662/724 HU, and the anteroposterior diameter, width, and cross-sectional area were measured by verifying on the axial cross-sectional image.

With respect to the PPS, the minimum width was measured to clarify the boundary with the nasal cavity, and the cross-sectional area was defined as the area surrounded by the minimum width and the anteroposterior diameter, especially. For the other four sites, the maximum anteroposterior diameter, width, and cross-sectional area were measured (Figure 2(b)). An additional measurement variable, the minimum area (MA), corresponding to the narrowest part of the airway size was determined; the anteroposterior diameter, width, and cross-sectional area of the MA were also measured (cm/cm^2^; Figure 3).

**Figure 4:**
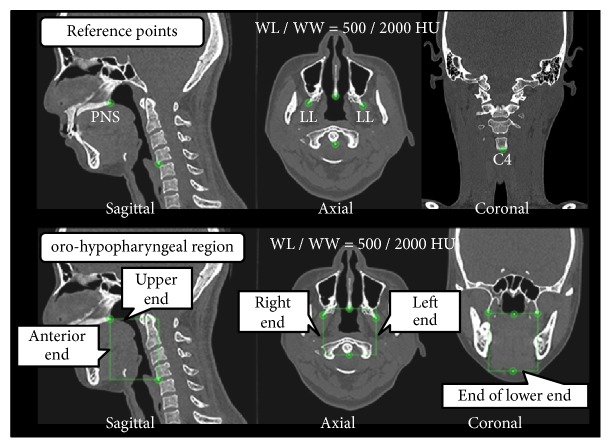
The oro-hypopharyngeal region on the multiplanar reconstruction (MPR) image. Abbreviation: WL/WW, window level/window width.

Additionally, steps were performed to reconstruct three-dimensional upper pharyngeal airway images for volumetric analysis.  Figure 4 depicts the method used to set the oro-hypopharyngeal region portion on the MPR image; four points were defined to set this region. The reference points of the anterior and upper ends were set as the PNS, the left and right ends and the lower end were set as the lamina lateralis of the pterygoid process (LL), and the lower end was set as C4 on the MPR image by verifying on the median sagittal, axial, and coronal cross-sectional images. The area surrounded by the reference points was set as the oro-hypopharyngeal region (WL/WW was set to the CT-bone value of 500/2000 HU).

**Figure 5:**
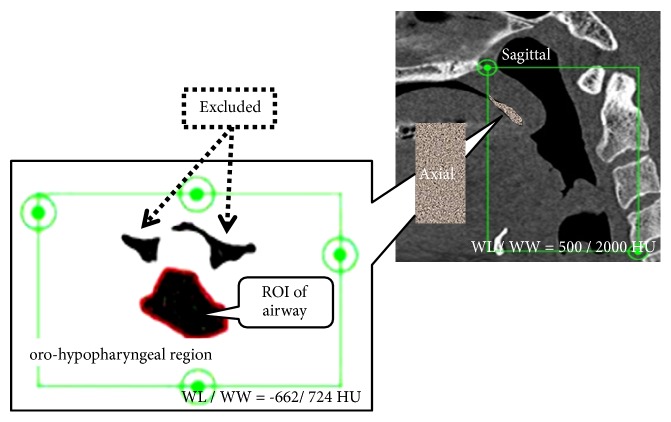
The region of interest (ROI) for calculating the airway volume. Abbreviations: WL/WW, window level/window width.

Figure 5 depicts the airway volume ROI. The ROI of the airway was set by manually tracing all axial cross-sectional images of the oro-hypopharyngeal region. To establish the ROI, the movable region of the soft palate was positioned as the oral cavity side and it was excluded from the setting range to clarify the boundary with the pharyngeal side.

**Figure 6:**
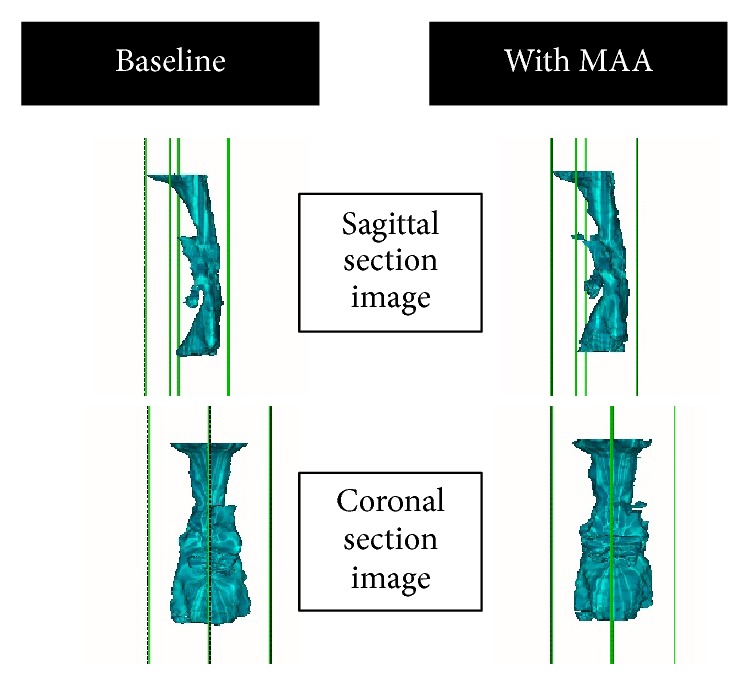
An example of a three-dimensional image of the upper airway. A three-dimensional surface rendering was constructed using all the airway regions of interest in the oro-hypopharyngeal region using multislice computed tomography data at baseline and data with the mandibular advancement appliance (MAA).

**Figure 7:**
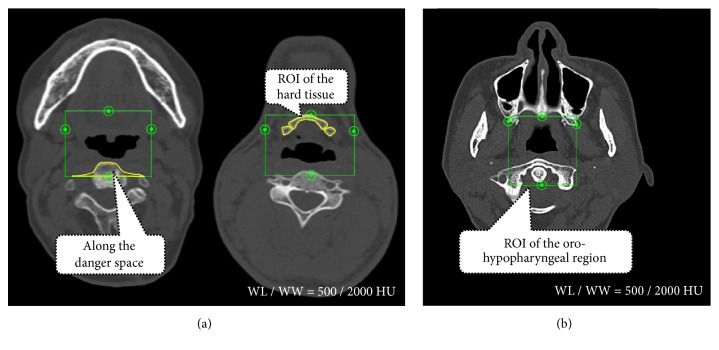
The region of interest (ROI) used for calculating the hard tissue and oro-hypopharyngeal region volumes. The ROI of the hard tissue and oro-hypopharyngeal region were set by manually tracing all the axial cross-sectional images of the oro-hypopharyngeal region. To set the ROI, a danger space was used in the area of the cervical vertebrae to maintain bone morphology continuity. All the hard tissue and oro-hypopharyngeal region ROIs in the oro-hypopharyngeal region assessed using multislice computed tomography data at baseline and data with the mandibular advancement appliance (MAA) were superimposed. Abbreviations: WL/WW, window level/window width.

Finally, the volumes of the upper airway, bony hard tissue (lower facial skeleton primarily including the hyoid and cervical vertebrae), and soft tissue (soft palate and tongue) in the oro-hypopharyngeal region were measured (cm^3^; Figures 6 and 7). The soft tissue volume, including the soft palate and tongue, was calculated by subtracting the total airway and hard tissue volumes from the oro-hypopharyngeal region volume. The soft tissue volume observed in the area of the oro-hypopharyngeal region was also evaluated. Additionally, the volumes of the airway, hard tissue, and soft tissue were divided by the oro-hypopharyngeal region volume to obtain the ratios for the oro-hypopharyngeal region (%).

### 2.5. Polysomnography

All patients were instructed to undergo a complete polysomnography (PSG) study both at baseline and after the follow-up period while wearing the MAA. For the PSG analyses, a Rembrandt (Koike Medical, Tokyo, Japan) and/or EMBLA N7000 (Chest corp., Tokyo, Japan) system was used by one sleep physician. The apnea index (AI), hypopnea index (HI), apnea-hypopnea index (AHI), and lowest arterial oxygen saturation (SpO_2_) were assessed. Apnea is defined as a decrease in airflow of more than 80% for at least 10 seconds, while hypopnea is defined as at least a 30% decrease in airflow with oxygen desaturation greater than or equal to 4%.

### 2.6. Statistical Analyses

The statistical analyses were conducted using a paired t-test to analyze the difference between two MPR images. Descriptive statistics consisted of analyzing the data average and standard deviation. A* P*-value of 0.05 was considered significant and a* P*-value of 0.01 was considered highly significant.

## 3. Results

Table 1 presents the evaluation of the PSG data. The AI, HI, and AHI significantly decreased following MAA therapy. Moreover, the lowest SpO_2_ was significantly higher than the baseline level.

**Figure 8:**
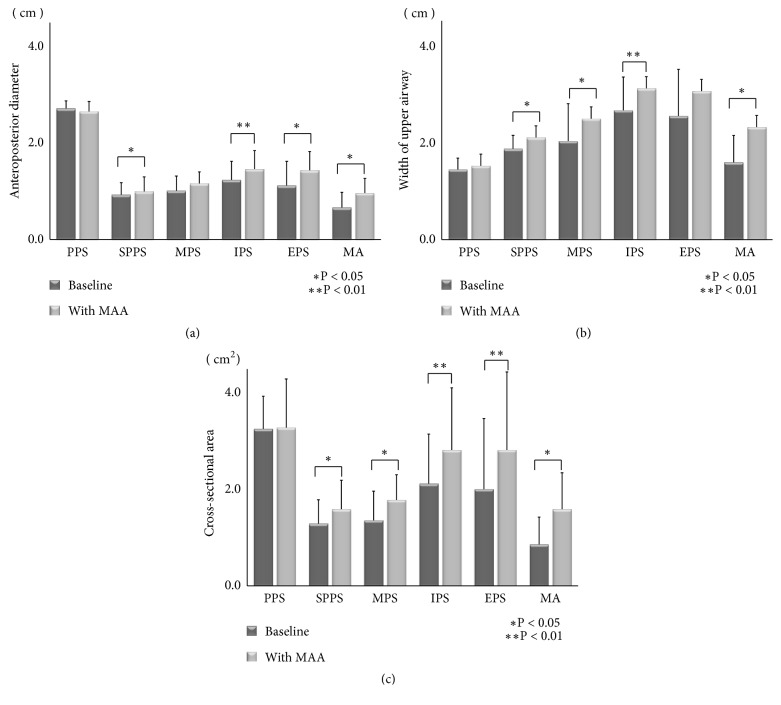
(a) Comparison of the anteroposterior diameters of the six sites between baseline and data with the mandibular advancement appliance (MAA). Abbreviations: EPS, epiglottic pharyngeal space; IPS, inferior pharyngeal space; MA, minimum area; MPS, middle pharyngeal space; PPS, palatal pharyngeal space; SPPS, superior posterior pharyngeal space. (b) Comparison of the widths of the six sites between baseline and data with the mandibular advancement appliance (MAA). Abbreviations: EPS, epiglottic pharyngeal space; IPS, inferior pharyngeal space; MA, minimum area; MPS, middle pharyngeal space; PPS, palatal pharyngeal space; SPPS, superior posterior pharyngeal space. (c) Comparison of the cross-sectional area of the six sites between baseline and data with the mandibular advancement appliance (MAA). Abbreviations: EPS, epiglottic pharyngeal space; IPS, inferior pharyngeal space; MA, minimum area; MPS, middle pharyngeal space; PPS, palatal pharyngeal space; SPPS, superior posterior pharyngeal space.

Comparison of the anteroposterior diameter from the MPR image data demonstrated that the SPPS, IPS, EPS, and MA significantly increased after MAA therapy. Conversely, the PPS and MPS did not significantly change (Figure 8(a)). Comparison of the width of the six measurements showed that the SPPS, MPS, IPS, and MA significantly increased after MAA therapy. Although the PPS and EPS demonstrated values higher than at baseline, statistical significance was not reached (Figure 8(b)). With respect to the cross-sectional area, all of the variables, except the PPS, significantly increased after MAA therapy (Figure 8(c)).

**Figure 9:**
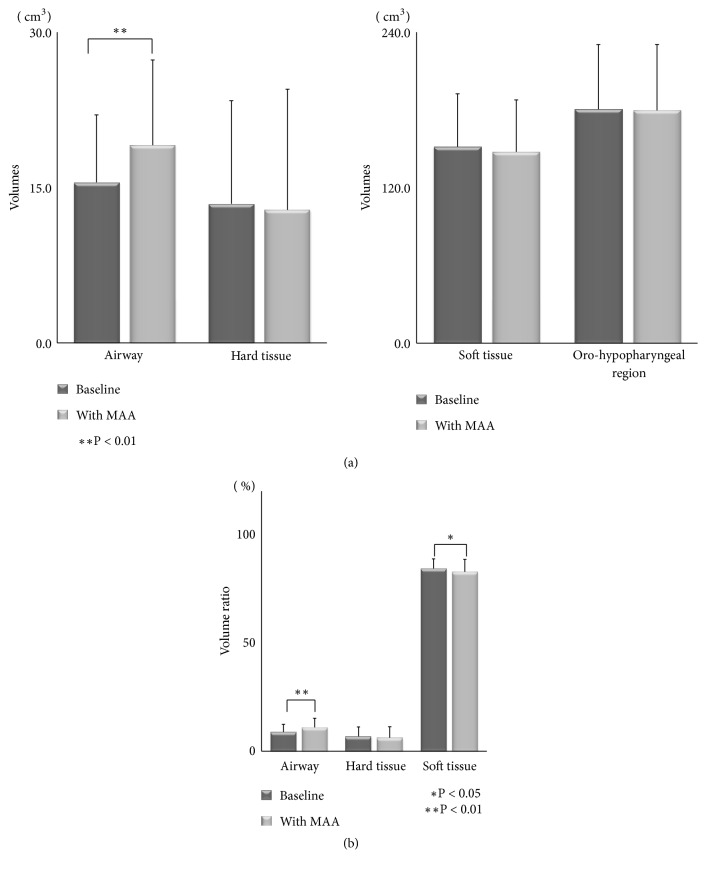
(a) Comparison of the airway, hard and soft tissue volumes, and oro-hypopharyngeal region between baseline and data with the mandibular advancement appliance (MAA). (b) Comparison of the ratios of the airway, and hard and soft tissue volumes in the oro-hypopharyngeal region between baseline and data with the mandibular advancement appliance (MAA).

In the volumetric analysis, a significant increase was observed in airway volume (mean volume: from 15.5 cm^3^ to 19.1 cm^3^), whereas the hard and oro-hypopharyngeal region volumes were nearly the same after MMA therapy. The volume of soft tissue in the oro-hypopharyngeal region showed the decrease, although the significance was not presented (Figure 9(a)). However, from a different point of view, the volumes of the upper airway and soft tissue significantly increased and decreased, respectively, as demonstrated by the calculated ratio for the oro-hypopharyngeal region (Figure 9(b)).

## 4. Discussion

This study quantitatively assessed the three-dimensional morphology of the pharyngeal airway and its changes in relation to MAA therapy in supine patients with OSA using MSCT.

Three-dimensional records provide the considerable advantage of viewing objects at their actual (100%) size without concern for distortion, magnification, or superimposed anatomical objects that are typical with plane film images [13]. Moreover, CT images can be reconstructed and used to quantitatively assess pharyngeal airway size using volumetric analysis. Previous CT studies have evaluated airway caliber with the subjects in the upright position [8–10]. Martin et al. [14] reported that a change in body position affects upper airway size. Patients with OSA exhibited smaller decreases in upper airway cross-sectional area when their body position changed from the upright to the supine position. The upper airway and surrounding soft tissue structures may be influenced by gravitational forces in the supine position during sleep; therefore, it is of great importance that MSCT data is conducted with OSA patients in the supine position.

In most of the assessed airway size variables, significant increases in the anteroposterior diameter and width were observed after MAA therapy. Regarding the upper airway cross-sectional area, all the upper airway size variables exhibited significant increases, except the PPS. Several studies have discussed the mechanism of action of MAA therapy, although the site of airway size enlargement was different in the various assessments [15–17]. Bennet et al. assumed that MAAs exert their effects predominantly in the oropharynx and hypopharynx, rather than the velopharynx (retropalatal area) [15]. In contrast, Lowe et al. reported that the effects of the appliance were observed in the velopharynx and the retroglossal area using video examination [16]. Schmit-Nowara et al. also found an increase in the velopharynx and retroglossal airspace with MAA use by using cephalometric analysis [17]. Liu et al. proposed two possible mechanisms of MAA therapy [18]. First, anterior movement of the tongue may decrease the gravitational effect on the soft palate. Second, forward displacement of the mandible may decrease the collapsibility of the velopharynx and retroglossal airspace in the oropharynx because the lateral wall of the soft palate anatomically connects to the base of the tongue through the palatoglossal arch, and mandibular advancement may stretch the soft palate via this mechanical connection, thereby stiffening the velopharyngeal segment.

In the present study, out of the six assessed variables, the SPPS, IPS, and MA most reflected the effects of MAA therapy on upper airway size, while the PPS was the least affected. It could be speculated that the reason the PPS did not exhibit any significant change is that it is the highest (i.e., uppermost) area of the upper airway, in the region of the nasopharynx. Based on these results, it could be concluded that MAA therapy primarily affects the velopharynx and oropharynx (and the surrounding soft palate and tongue).

In our volumetric analysis, the reconstructed airway volume significantly increased after MAA therapy. Interestingly, the ratios of the airway and soft tissue against the oro-hypopharyngeal region were significantly increased and decreased, respectively, after MAA therapy. The fact that the ratio of the soft tissue volume in the oro-hypopharyngeal region was less could be of significance for improving OSA. We measured the soft tissue volume that induces pressure on and collapsibility of the upper airway, which was observed by MSCT in the experimental area. It might not be critical to evaluate the full net volume of the soft tissue. In our opinion, the anatomical balance is more important; this means that not just the airway size, but also the surrounding hard and soft tissue volume is involved in OSA. That is the reason why we calculated and evaluated the ratio of soft tissue volume against the oro-hypopharyngeal region.

This study had limitations, including its small sample size and the fact that the patients were awake during the examination. The MSCT data techniques during sleep could provide additional information about the mechanism of action of the MAA. However, that method is limited to research conditions and might be impractical in a large group of patients. To understand the pathogenesis of OSA and the clinical applicability of MAA fully, further investigations that focus on three-dimensional airway enlargement analysis of various sites that are affected by MAA therapy are required in a larger number of patients with OSA.

## 5. Conclusions

We demonstrated that the proportional size of the soft tissue volume, i.e., the soft palate and tongue in the oro-hypopharyngeal region, significantly decreased during use of an MAA. This forward displacement of the soft tissue thereby increases the retroglossal airway space (except the nasopharynx) three-dimensionally.

## Figures and Tables

**Table 1:** Evaluation of the polysomnography data.

Variables	baseline	follow-up period	P-value
AI (events/h)	3.4±4.3	0.4±0.3	*∗*
HI (events/h)	11.3±5.2	5.2±3.3	*∗∗*
AHI (events/h)	14.7±4.9	5.6±3.3	*∗∗*
Lowest SpO_2_ (%)	87.7±7.7	90.9±3.7	*∗*

Abbreviations: AHI, apnea-hypopnea index; AI, apnea index; HI, hypopnea index; SpO_2_, arterial oxygen saturation. *∗*P < 0.05, *∗∗*P < 0.01.

## Data Availability

The data used to support the findings of this study are available from the corresponding author upon request.

## Appendix

MAA: Mandibular advancement appliance
MSCT: Multislice computed tomography
CBCT: Cone-beam computed tomography
ROI: Region of interest
MPR: Multiplanar reconstruction
PPS: Palatal pharyngeal space
SPPS: Superior posterior pharyngeal space
MPS: Middle pharyngeal space
IPS: Inferior pharyngeal space
EPS: Epiglottic pharyngeal space
PNS: Posterior nasal spine
LL: Lamina lateralis of the pterygoid process
MA: Minimum area
WL: Window level
WW: Window width.
